# P031. An observational study on chronic tension-type headache treatment with Quantum Molecular Resonance according to I.A.R.A. model^®^

**DOI:** 10.1186/1129-2377-16-S1-A176

**Published:** 2015-09-28

**Authors:** Francesca Gulotta, Licia Grazzi, Giovanni B Allais, Sara Rolando, Maria G Saracco, Maurizio Cavallini, Andrea De Giorgio, Anna M Padovan, Stefania Pelosin, Paolo Agagliati, Marco Aguggia

**Affiliations:** Kiara Association, Turin, Italy; Neurological Institute “C. Besta”, Milan, Italy; Department of Surgical Sciences, Woman's Headache Center, University of Turin, Turin, Italy; SOC Neurology Cardinal Massaia Hospital, Asti, Italy; eCampus University, Novedrate, CO Italy

## Introduction

Chronic tension-type headache (CTTH) is a clinical entity where high muscle tension level, in particular in the trapezium area, may induce a pain sensation in the same area. Quantum Molecular Resonance (QMR) has been proved to promote cell regeneration process through direct cell stimulation which is able to decrease local inflammatory reaction and consequently pain level. This study reports a clinical experience of QMR treatment for tension-type headache, associated to I.A.R.A. (Incontro, Alleanza, Responsabilità, Autonomia) model^**®**^, which increases consciousness of patients who can participate actively to QMR therapy. Treatment has been administrated by specialised nurses.

## Materials and methods

From March 2014 to May 2015 a group of 40 patients, (33 females/7 males), suffering from CTTH, diagnosed according to the IHS criteria, underwent 8 sessions of QMR treatment protocol, 2 treatments per week, lasting 20 minutes each. During treatment 3 female patients withdrew their informed consent. QMR technique consists of applying 2 electrodes on the lower trapezius area, 1 electrode in the median part of the trapezius and a probe administration on the median trapezius area and on the forehead region. A prophylactic treatment for CTTH (antidepressants and/or muscle relaxants) was used by 89.2% of patients, 24.3% used a symptomatic and 13.5% both. They recorded headache episodes and medication intake in a daily diary. Follow-up meetings were fixed at 1, 3, 6 months after the end of the program.

## Results

**Figure 1:**
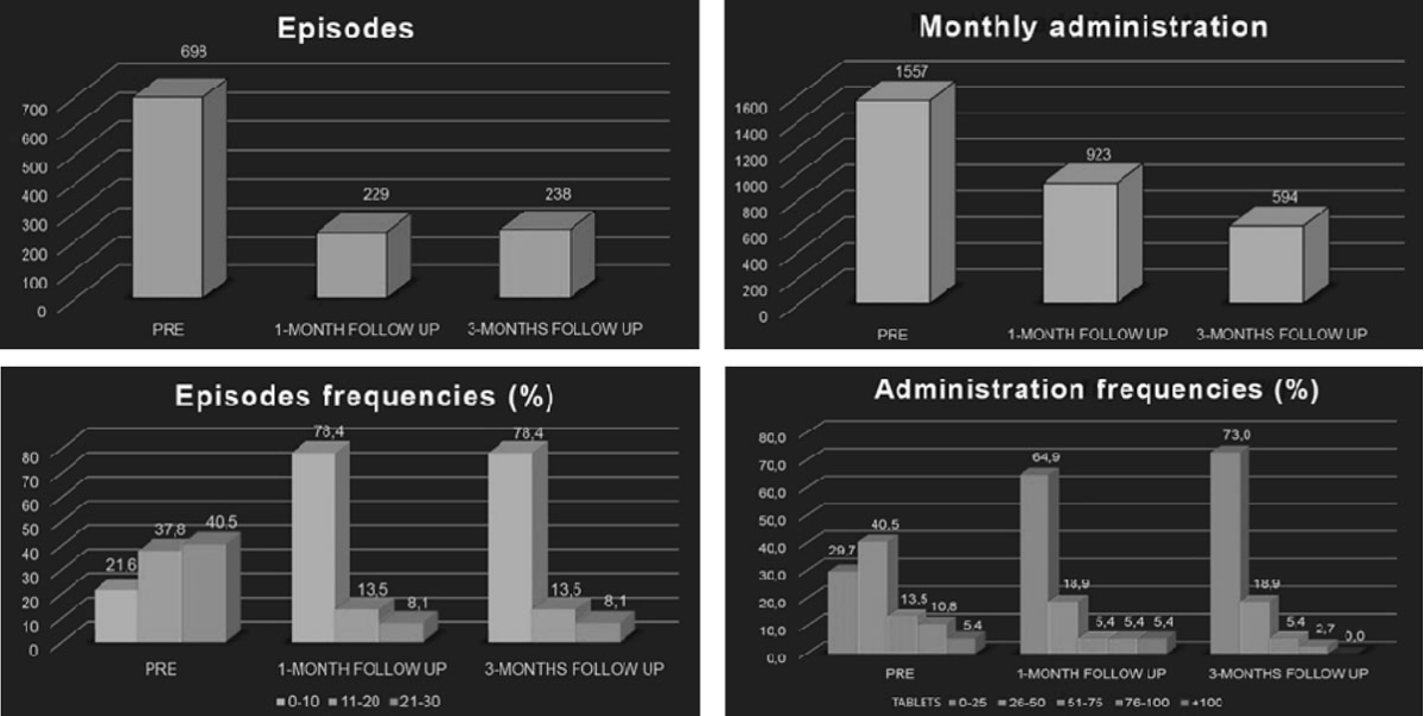
Total number and frequencies of headache episodes and drug administration.

Days of headache/month decreased significantly from 19±9.5 before treatment to 6±8.4 at 1-month follow-up (p < 0.001) and to 6±8.6 at 3-month follow-up (p<0.001) (Figure 1). Patients did not report any side effects.

## Conclusions

QMR seems to be effective for patients with CTTH and results are confirmed until the 3-month follow-up. Treatment is well tolerated and safe for patients. Further studies and longer follow-up will be necessary to confirm the efficacy of this innovative approach.

Written informed consent to publish was obtained from the patient(s).

